# Synthesis, crystal structure and photophysical properties of chlorido­[(*E*)-3-hy­droxy-2-methyl-6-(quinolin-8-yldiazen­yl)phenolato]copper(II) monohydrate

**DOI:** 10.1107/S2056989022003437

**Published:** 2022-04-05

**Authors:** Chihiro Kachi-Terajima, Seiya Hagiwara

**Affiliations:** a Department of Chemistry, Faculty of Science, Toho University, 2-2-1 Miyama, Funabashi, Chiba 274-8510, Japan

**Keywords:** crystal structure, copper(II) complex, quinoline-based azo ligand, electronic absorption spectra

## Abstract

The reaction between copper(II) chloride dihydrate and the (*E*)-2-methyl-4-(quinolin-8-yldiazen­yl)benzene-1,3-diol ligand in aceto­nitrile leads to the formation of the title compound, [Cu(C_16_H_12_N_3_O_2_)Cl]·H_2_O. The ligand is deprotonated and coordinates with three donor atoms (tridentate) to the Cu^II^ ion. Individual mol­ecules of the Cu^II^ complex are connected by chloride bridges, forming a one-dimensional coordination polymer. No photoisomerization to the *cis* isomer of the azo ligand was observed upon irradiation with UV light.

## Abstract 2

A copper(II) complex with the (*E*)-2-methyl-4-(quinolin-8-yldiazen­yl)benzene-1,3-diol ligand was prepared and structurally characterized. The UV–Vis absorption spectra of the ligand and the complex are reported.

## 1. Chemical context

Azo­benzene derivatives are well-known dyes with fascinating characteristics such as *cis–trans* photoisomerization and azo–hydrazone tautomerism. The combination of azo compounds with metal ions to form complexes is a promising approach for controlling their photophysical properties. In metal complexes with azo ligands, the metal centers and azo ligands can affect each other’s properties. For example, *cis–trans* photoisomerization by irradiation with a single frequency of light has been achieved in azo-conjugated metal complexes by a combination of the photophysical and the redox properties of ligand and metal center (Nishihara, 2005 ▸). Azo­benzene deriv­atives with hy­droxy groups in the *ortho* or *para* position tend to form hydrazone tautomers (Jacques *et al.*, 1979 ▸; Ball & Nicholls, 1982 ▸; Rauf *et al.*, 2015 ▸). A hydrazone tautomer can be converted to an azo tautomer by complexation to the metal ion (Chen *et al.*, 2012 ▸; Cai *et al.*, 2016 ▸). In this study, we used the *ortho* and *para* isomer of the hy­droxy-substituted azo­benzene derivative, (*E*)-2-methyl-4-(quinolin-8-yldiazen­yl)benz­ene-1,3-diol, to investigate azo–hydrazone tautomerism in its Cu^II^ complex. The photophysical properties of the ligand and the Cu^II^ complex were studied by UV–Vis spectroscopy to address the potential photoisomerization. 
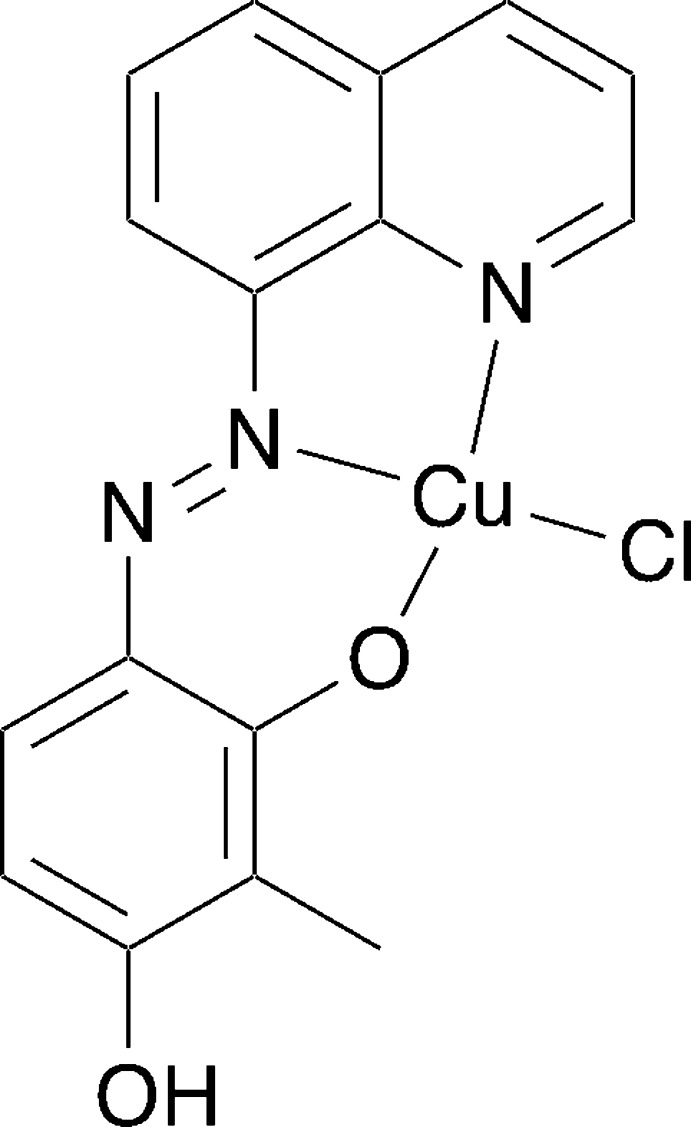


## 2. Structural commentary

**Figure 1:**
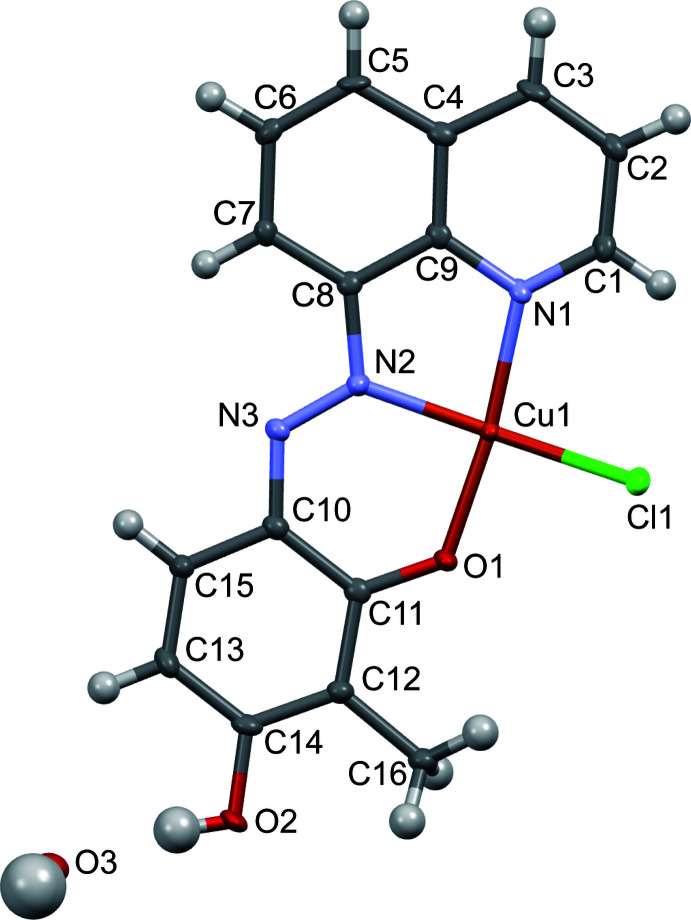
Crystal structure of the title compound showing the atom-labeling scheme, generated with *Mercury* software (Version 2021.2.0; Macrae *et al.*, 2020 ▸). Displacement ellipsoids are drawn at the 50% probability level.

The crystal structure of the Cu^II^ complex is shown in Fig. 1 ▸. The asymmetric unit contains one Cu^II^ complex and one solvent water mol­ecule. The hy­droxy group in the *ortho*-position of the azo ligand is deprotonated and is coordinated the Cu^II^ center. In the asymmetric unit, the Cu^II^ ion is 4-coordinated in a distorted square-planar geometry. The donor atoms comprise one nitro­gen atom of the quinoline moiety, one nitro­gen atom of the azo group, one deprotonated alcohol oxygen atom, and a chloride ion. The other hy­droxy group of the azo ligand, in the *para*-position, remains protonated. The chlorido ligand is also weakly coordinated by an adjacent Cu^II^ center occupying its apical position, resulting in an elongated square-pyramidal coordination polyhedron around the copper(II) ions. The Cu1—Cl1^i^ distance in the apical position is 2.7395 (10) Å, which is notably longer than the distances in the equatorial positions, Cu1—Cl1 = 2.2803 (8) Å, Cu1—O1 = 1.917 (2) Å, Cu1—N1 = 2.008 (3) Å, and Cu1—N2 = 1.945 (3) Å [symmetry code: (i) *x* + 1, *y*, *z*]. The N2—N3 bond distance of 1.293 (4) Å is typical for the N=N double bond of an azo group. The structural features of the aromatic rings and the C11—O1 single-bond length of 1.300 (4) Å also indicate that the ligand adopts the azo structure, rather than the hydrazone structure, which is similar to the structures observed in analogous azo-metal complexes with other metals, including Ni, Cu, and Zn (Cai *et al.*, 2016 ▸; Kochem *et al.*, 2011 ▸, 2014 ▸).

## 3. Supra­molecular features

**Figure 2:**
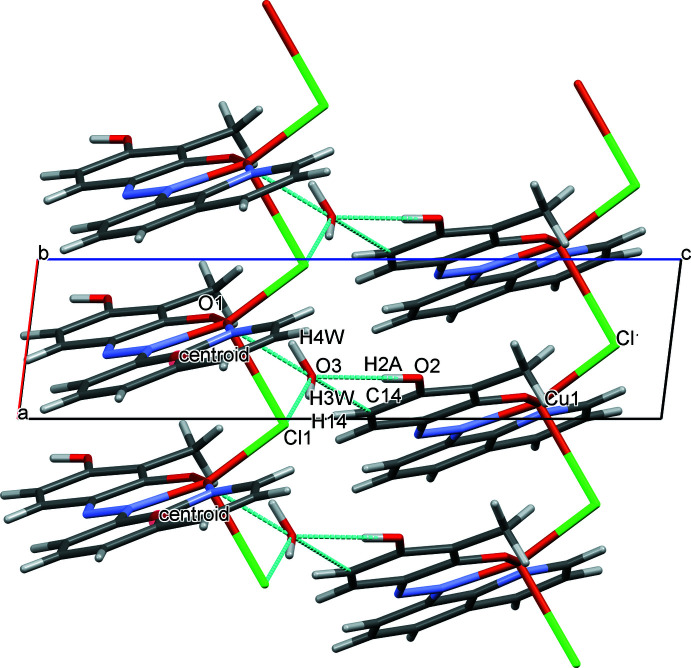
Crystal packing of the title compound viewed along the *b* axis showing inter­molecular hydrogen bonds and π–π stacking between the azo ligands, generated with *Mercury* software (Version 2021.2.0; Macrae *et al.*, 2020 ▸). Inter­molecular hydrogen bonds are shown as blue dashed lines.

**Figure 3:**
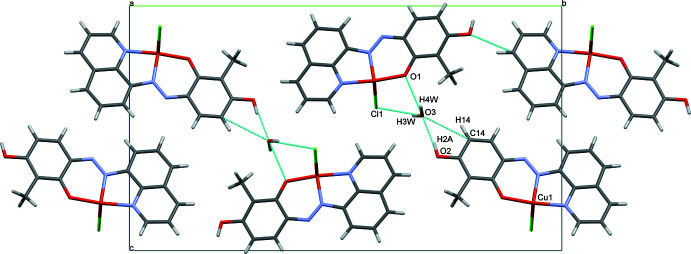
Crystal packing of the title compound viewed along the *a* axis showing inter­molecular hydrogen bonds (blue dashed lines), generated with *Mercury* software (Version 2021.2.0; Macrae *et al.*, 2020 ▸).

The coordinated chloride ion bridges adjacent Cu^II^ complexes to form a one-dimensional coordination polymer resulting in columns along the crystallographic *a-*axis direction (Fig. 2 ▸). This is supported by π–π stacking between the co-planar quinoline rings with a centroid–centroid distance of 3.7711 (4) Å, an inter-plane distance of 3.3494 (12) Å, and a slippage of 1.733 (2) Å. The 1D columns are linked through hydrogen bonds facilitated by the solvent water mol­ecules, C14—H14⋯O3, O2—H2*A*⋯O3, O3—H3*W*⋯Cl1^i^, and O3—H4*W*⋯O1^ii^, [symmetry codes: (i) *x* + 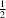 , −*y* + 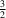 , *z* − 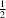 ; (ii) *x* − 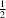 , −*y* + 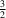 , *z* − 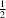 ] (Table 1 ▸, Fig. 3 ▸).

## 4. Database survey

A search of the Cambridge Structural Database (CSD Version 5.42, update of November 2020; Groom *et al.*, 2016 ▸) with *ConQuest* (Version 2020.3.0; Bruno *et al.*, 2002 ▸) for phenyl- and quinolinyl-bearing azo ligands with an *ortho-*hy­droxy substituent and their complexes resulted in only seven hits. These structures include one ligand derivative and six trans­ition-metal complexes (an azo­benzene derivative and its Zn complex, refcodes ONOKUY and ONOLAF; Kochem *et al.*, 2011 ▸; Cu complexes, refcodes MOGLAX and MOGLEB; Kochem *et al.*, 2014 ▸; an Re complex, refcode TOZTUZ; Sarkar *et al.*, 2015 ▸; a Co complex, refcode VARQUD; Taylor *et al.*, 2017 ▸; an Ho complex, refcode NAMJIY; Taylor *et al.*, 2018 ▸). While co-planarity of the aromatic moieties was observed in some of these structures, the formation of the column-type coordination polymeric structure of the title compound has no precedence in this group.

## 5. UV–Vis spectra for the azo ligand and Cu^II^ complex

**Figure 4:**
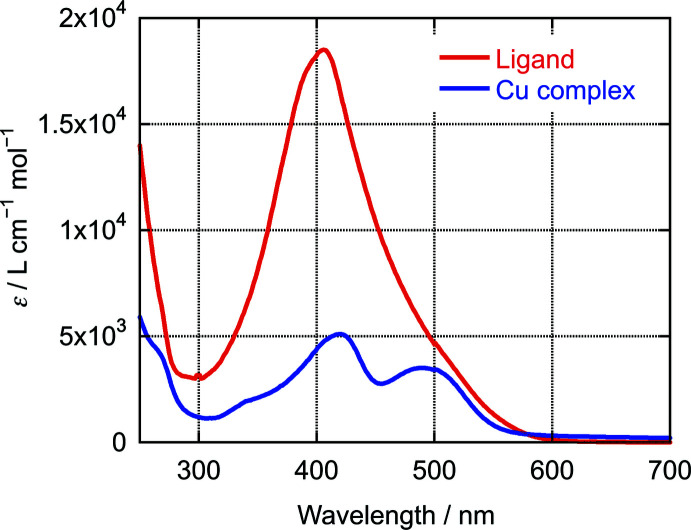
UV–Vis spectra of the ligand and the title compound in CH_3_CN.

The UV–Vis spectra of the azo ligand and the Cu^II^ complex in CH_3_CN are shown in Fig. 4 ▸. The maximum of the extinction (*ɛ*_max_) was observed at 406 nm for the ligand, while the Cu^II^ complex showed decreased absorption and red-shifted maxima at 420 and 489 nm. To investigate the photoisomerization of the ligand and the Cu^II^ complex, the samples were irradiated at maximum wavelength, but no photoisomerization to the *cis* isomer was observed for either compound.

## 6. Synthesis and crystallization

To synthesize the title ligand, an aqueous solution of 1.2 *M* NaNO_2_ (3 mL) was slowly added to a cold solution of 8-amino­quinoline (0.432 g, 3.00 mmol) in 0.5 *M* HCl_(aq)_ (20 mL). The resulting solution was stirred at 277 K for 15 min, and an aqueous solution of (NH_2_)_2_CO (0.180 g, 3.00 mmol) in 3 mL of water was then added to give a diazo­nium chloride solution. This solution was added to an aqueous 0.25 *M* NaOH solution of 2,6-di­hydroxy­toluene (0.372 g, 3.00 mmol) and stirred at 277 K for 30 min and then stirred at room temperature for 15 h. The reaction mixture was acidified with 1 *M* HCl(aq) (10 mL) and a red precipitate was formed. The precipitate was filtered off and washed with water and then with cold tetra­hydro­furan. Yield, 86% (0.787 g, 2.58 mmol). IR: *ν*_max_ (KBr): 3400, 3068, 1633, 1536, 1503, 1488, 1447, 1364, 1299, 1212, 787 cm^−1. 1^H NMR (400 MHz, CD_3_CN): *δ*_H_ 9.06 (*d*, 1H), 8.41 (*d*, 1H), 8.20 (*d*, 1H), 7.97 (*d*, 1H), 7.74 (*t*, 1H), 7.62 (*dd*, 1H), 7.49 (*d*, 1H), 6.63 (*d*, 1H), 2.12 (*s*, 3H). Analysis calculated for C_16_H_13_N_3_O_2_·0.72HCl: C, 62.90; H, 4.53; N, 13.75. Found: C, 62.49; H, 4.31; N, 14.17. The Cu^II^ complex was obtained as a brown solid by the reaction of the azo ligand synthesized as described above (0.099 g, 0.324 mmol) in 4 mL of ethanol with CuCl_2_·2H_2_O (0.061 g, 0.358 mmol) in 2 mL of H_2_O. Yield, 54% (0.073 g, 0.193 mmol). Crystals of the Cu^II^ complex suitable for the X-ray crystallography study were obtained by the slow diffusion of a CH_3_CN solution of the ligand into an aqueous solution of CuCl_2_·2H_2_O. IR: *ν*_max_ (KBr): 3418, 2924, 2854, 1633, 1557, 1508, 1436, 1283, 1258, 1048 cm^−1^. Analysis calculated for C_16_H_12_ClCuN_3_O_2_: C, 50.94; H, 3.21; N, 11.14. Found: C, 50.82; H, 3.63; N, 11.49.

## 7. Refinement

Crystal data, data collection and structure refinement details are summarized in Table 2 ▸. All non-hydrogen atoms were refined anisotropically. The O—H hydrogen atoms of the solvent water mol­ecules and the hy­droxy group in the *para*-position were found in the difference-Fourier map and were refined isotropically without restraints or constraints. Other hydrogen atoms were generated geometrically, and refined with a riding model with C—H = 0.98 Å, *U*_iso_(H) = 1.5 *U*_eq_(C) for methyl, and C—H = 0.95 Å, *U*_iso_(H) = 1.2 *U*_eq_(C) for aromatic hydrogen atoms. Two reflections were omitted as clear outliers.

## Supplementary Material

Crystal structure: contains datablock(s) I. DOI: 10.1107/S2056989022003437/yz2018sup1.cif

Structure factors: contains datablock(s) I. DOI: 10.1107/S2056989022003437/yz2018Isup2.hkl

CCDC reference: 2162331

Additional supporting information:  crystallographic information; 3D view; checkCIF report

## Figures and Tables

**Table 1:** Hydrogen-bond geometry (Å, °)

*D*—H⋯*A*	*D*—H	H⋯*A*	*D*⋯*A*	*D*—H⋯*A*
C14—H14⋯O3	0.95	2.65	3.318 (4)	128
O2—H2*A*⋯O3	0.82 (4)	1.87 (4)	2.686 (4)	172 (4)
O3—H3*W*⋯Cl1^i^	0.70 (4)	2.45 (4)	3.104 (3)	157 (5)
O3—H4*W*⋯O1^ii^	0.89 (6)	2.20 (6)	2.911 (4)	136 (4)

Symmetry codes: (i) 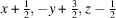 ; (ii) 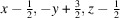 .

**Table 2:** Experimental details

Crystal data	
Chemical formula	[Cu(C_16_H_12_N_3_O_2_)Cl]·H_2_O
*M*_r_	395.29
Crystal system, space group	Monoclinic, *P*2_1_/*n*
Temperature (K)	100
*a*, *b*, *c* (Å)	3.7711 (4), 26.451 (3), 15.0864 (15)
β (°)	97.100 (2)
*V* (Å^3^)	1493.3 (3)
*Z*	4
Radiation type	Mo *K*α
μ (mm^−1^)	1.66
Crystal size (mm)	0.44 × 0.09 × 0.02

Data collection	
Diffractometer	Bruker APEXII CCD
Absorption correction	Multi-scan (*SADABS*; Krause *et al.*, 2015 ▸)
*T*_min_, *T*_max_	0.629, 0.745
No. of measured, independent and observed [*I* > 2σ(*I*)] reflections	8133, 2746, 2132
*R*_int_	0.049
(sin θ/λ)_max_ (Å^−1^)	0.602

Refinement	
*R*[*F* ^2^ > 2σ(*F* ^2^)], *wR*(*F* ^2^), *S*	0.036, 0.081, 1.04
No. of reflections	2746
No. of parameters	230
H-atom treatment	H atoms treated by a mixture of independent and constrained refinement
Δρ_max_, Δρ_min_ (e Å^−3^)	0.42, −0.41

Computer programs: *APEX2* and *SAINT* (Bruker, 2014 ▸), *SHELXT2014/5* (Sheldrick, 2015*a* ▸), *SHELXL2017/1* (Sheldrick, 2015*b* ▸), and *SHELXTL* (Sheldrick, 2008 ▸).

## supplementary crystallographic information

### Crystal data

**Table:**

[Cu(C_16_H_12_N_3_O_2_)Cl]·H_2_O	*F*(000) = 804
*M**_r_* = 395.29	*D*_x_ = 1.758 Mg m^−^^3^
Monoclinic, *P*2_1_/*n*	Mo *K*α radiation, λ = 0.71073 Å
*a* = 3.7711 (4) Å	Cell parameters from 1510 reflections
*b* = 26.451 (3) Å	θ = 2.7–24.3°
*c* = 15.0864 (15) Å	µ = 1.66 mm^−^^1^
β = 97.100 (2)°	*T* = 100 K
*V* = 1493.3 (3) Å^3^	Plate, brown
*Z* = 4	0.44 × 0.09 × 0.02 mm

### Data collection

**Table:**

Bruker APEXII CCD diffractometer	2132 reflections with *I* > 2σ(*I*)
φ and ω scans	*R*_int_ = 0.049
Absorption correction: multi-scan (SADABS; Krause *et al.*, 2015)	θ_max_ = 25.4°, θ_min_ = 2.1°
*T*_min_ = 0.629, *T*_max_ = 0.745	*h* = −4→4
8133 measured reflections	*k* = −21→31
2746 independent reflections	*l* = −17→18

### Refinement

**Table:**

Refinement on *F*^2^	Primary atom site location: dual
Least-squares matrix: full	Hydrogen site location: mixed
*R*[*F*^2^ > 2σ(*F*^2^)] = 0.036	H atoms treated by a mixture of independent and constrained refinement
*wR*(*F*^2^) = 0.081	*w* = 1/[σ^2^(*F*_o_^2^) + (0.0286*P*)^2^ + 0.6665*P*] where *P* = (*F*_o_^2^ + 2*F*_c_^2^)/3
*S* = 1.04	(Δ/σ)_max_ = 0.001
2746 reflections	Δρ_max_ = 0.42 e Å^−^^3^
230 parameters	Δρ_min_ = −0.41 e Å^−^^3^
0 restraints	

### Special details

**Table:**

Geometry. All esds (except the esd in the dihedral angle between two l.s. planes) are estimated using the full covariance matrix. The cell esds are taken into account individually in the estimation of esds in distances, angles and torsion angles; correlations between esds in cell parameters are only used when they are defined by crystal symmetry. An approximate (isotropic) treatment of cell esds is used for estimating esds involving l.s. planes.

### Fractional atomic coordinates and isotropic or equivalent isotropic displacement parameters (Å^2^)

**Table:**

	*x*	*y*	*z*	*U*_iso_*/*U*_eq_	
C1	0.9143 (9)	1.03984 (12)	0.8827 (2)	0.0157 (7)	
H1	0.822246	1.026099	0.933371	0.019*	
C2	0.9909 (9)	1.09143 (12)	0.8821 (2)	0.0162 (7)	
H2	0.951775	1.111932	0.931701	0.019*	
C3	1.1222 (9)	1.11240 (12)	0.8101 (2)	0.0176 (8)	
H3	1.174056	1.147528	0.809169	0.021*	
C4	1.1803 (9)	1.08129 (12)	0.7369 (2)	0.0146 (7)	
C5	1.3129 (9)	1.09847 (12)	0.6585 (2)	0.0156 (8)	
H5	1.371662	1.133133	0.652668	0.019*	
C6	1.3564 (8)	1.06562 (12)	0.5914 (2)	0.0154 (7)	
H6	1.440700	1.077948	0.538699	0.018*	
C7	1.2798 (8)	1.01391 (12)	0.5981 (2)	0.0140 (7)	
H7	1.314425	0.991581	0.550631	0.017*	
C8	1.1548 (8)	0.99580 (12)	0.6736 (2)	0.0124 (7)	
C9	1.0988 (8)	1.02935 (12)	0.7434 (2)	0.0127 (7)	
C10	1.0113 (9)	0.86492 (12)	0.6316 (2)	0.0140 (7)	
C11	0.8938 (8)	0.83965 (12)	0.7082 (2)	0.0132 (7)	
C12	0.8113 (9)	0.78725 (12)	0.7008 (2)	0.0151 (7)	
C13	0.8379 (9)	0.76272 (12)	0.6209 (2)	0.0164 (8)	
C14	0.9591 (9)	0.78717 (13)	0.5468 (2)	0.0184 (8)	
H14	0.979522	0.768954	0.493360	0.022*	
C15	1.0453 (9)	0.83666 (12)	0.5529 (2)	0.0160 (7)	
H15	1.130421	0.852982	0.503555	0.019*	
C16	0.6924 (9)	0.75947 (12)	0.7792 (2)	0.0180 (8)	
H16A	0.534473	0.731513	0.757445	0.027*	
H16B	0.563395	0.782752	0.814287	0.027*	
H16C	0.901857	0.746056	0.816731	0.027*	
Cl1	0.5361 (2)	0.92919 (3)	0.91540 (5)	0.0160 (2)	
Cu1	0.90089 (11)	0.93390 (2)	0.80589 (2)	0.01287 (13)	
H2A	0.747 (11)	0.7051 (16)	0.563 (3)	0.045 (14)*	
H3W	0.853 (12)	0.6570 (17)	0.448 (3)	0.043 (18)*	
H4W	0.533 (15)	0.674 (2)	0.412 (4)	0.09 (2)*	
N1	0.9636 (7)	1.00915 (10)	0.81610 (16)	0.0126 (6)	
N2	1.0654 (7)	0.94509 (10)	0.69027 (17)	0.0141 (6)	
N3	1.0982 (7)	0.91430 (10)	0.62523 (17)	0.0131 (6)	
O1	0.8645 (6)	0.86267 (8)	0.78313 (14)	0.0157 (5)	
O2	0.7473 (7)	0.71334 (9)	0.61565 (17)	0.0225 (6)	
O3	0.7360 (10)	0.67746 (11)	0.44880 (19)	0.0275 (7)	

### Atomic displacement parameters (Å^2^)

**Table:**

	*U*^11^	*U*^22^	*U*^33^	*U*^12^	*U*^13^	*U*^23^
C1	0.0175 (19)	0.0184 (18)	0.0110 (16)	0.0034 (15)	0.0011 (14)	0.0007 (15)
C2	0.0185 (19)	0.0147 (18)	0.0144 (17)	0.0026 (15)	−0.0016 (15)	−0.0033 (15)
C3	0.023 (2)	0.0077 (17)	0.0206 (18)	0.0023 (14)	−0.0033 (15)	−0.0017 (15)
C4	0.0135 (18)	0.0114 (17)	0.0180 (17)	0.0024 (14)	−0.0012 (15)	0.0000 (14)
C5	0.0162 (19)	0.0076 (17)	0.0223 (19)	−0.0023 (14)	−0.0001 (15)	0.0044 (15)
C6	0.0148 (18)	0.0175 (18)	0.0136 (16)	−0.0015 (15)	0.0006 (14)	0.0045 (15)
C7	0.0126 (17)	0.0162 (18)	0.0136 (16)	0.0003 (14)	0.0029 (14)	−0.0019 (15)
C8	0.0114 (17)	0.0126 (17)	0.0131 (16)	0.0020 (14)	0.0004 (13)	0.0018 (14)
C9	0.0106 (17)	0.0144 (18)	0.0122 (16)	0.0017 (13)	−0.0027 (14)	−0.0006 (14)
C10	0.0137 (18)	0.0123 (17)	0.0160 (17)	0.0007 (14)	0.0010 (14)	−0.0014 (14)
C11	0.0107 (17)	0.0137 (17)	0.0145 (16)	0.0017 (14)	−0.0011 (14)	0.0000 (14)
C12	0.0168 (19)	0.0120 (17)	0.0168 (17)	−0.0014 (14)	0.0029 (14)	−0.0002 (15)
C13	0.020 (2)	0.0086 (17)	0.0205 (18)	0.0002 (14)	0.0003 (15)	−0.0002 (15)
C14	0.025 (2)	0.0156 (18)	0.0142 (17)	0.0045 (15)	0.0025 (15)	−0.0033 (15)
C15	0.0218 (19)	0.0124 (18)	0.0142 (16)	−0.0021 (15)	0.0043 (15)	−0.0009 (15)
C16	0.022 (2)	0.0126 (17)	0.0196 (18)	−0.0015 (15)	0.0036 (16)	−0.0004 (15)
Cl1	0.0190 (4)	0.0161 (4)	0.0137 (4)	0.0011 (4)	0.0049 (3)	0.0009 (3)
Cu1	0.0193 (2)	0.0089 (2)	0.0110 (2)	−0.00015 (17)	0.00432 (16)	−0.00005 (17)
N1	0.0161 (15)	0.0102 (14)	0.0112 (13)	0.0040 (12)	0.0011 (12)	−0.0001 (12)
N2	0.0178 (16)	0.0110 (14)	0.0141 (14)	−0.0007 (12)	0.0046 (12)	0.0001 (12)
N3	0.0165 (16)	0.0110 (14)	0.0115 (14)	−0.0005 (12)	0.0004 (12)	−0.0012 (12)
O1	0.0262 (14)	0.0087 (11)	0.0130 (11)	−0.0012 (10)	0.0051 (10)	−0.0013 (10)
O2	0.0403 (17)	0.0093 (13)	0.0195 (14)	−0.0043 (11)	0.0095 (12)	−0.0056 (11)
O3	0.040 (2)	0.0172 (15)	0.0252 (15)	−0.0017 (15)	0.0047 (15)	−0.0014 (13)

### Geometric parameters (Å, º)

**Table:**

C1—N1	1.323 (4)	C11—O1	1.300 (4)
C1—C2	1.395 (5)	C11—C12	1.422 (4)
C1—H1	0.9500	C12—C13	1.383 (4)
C2—C3	1.366 (4)	C12—C16	1.506 (4)
C2—H2	0.9500	C13—O2	1.350 (4)
C3—C4	1.416 (4)	C13—C14	1.417 (4)
C3—H3	0.9500	C14—C15	1.349 (4)
C4—C9	1.414 (4)	C14—H14	0.9500
C4—C5	1.415 (4)	C15—H15	0.9500
C5—C6	1.359 (4)	C16—H16A	0.9800
C5—H5	0.9500	C16—H16B	0.9800
C6—C7	1.404 (4)	C16—H16C	0.9800
C6—H6	0.9500	Cl1—Cu1	2.2803 (8)
C7—C8	1.372 (4)	Cl1—Cu1^i^	2.7395 (10)
C7—H7	0.9500	Cu1—O1	1.917 (2)
C8—C9	1.412 (4)	Cu1—N2	1.945 (2)
C8—N2	1.413 (4)	Cu1—N1	2.008 (3)
C9—N1	1.374 (4)	N2—N3	1.293 (3)
C10—N3	1.353 (4)	O2—H2A	0.82 (4)
C10—C15	1.423 (4)	O3—H3W	0.70 (4)
C10—C11	1.451 (4)	O3—H4W	0.89 (6)
			
N1—C1—C2	123.2 (3)	O2—C13—C12	117.4 (3)
N1—C1—H1	118.4	O2—C13—C14	119.9 (3)
C2—C1—H1	118.4	C12—C13—C14	122.6 (3)
C3—C2—C1	119.9 (3)	C15—C14—C13	119.2 (3)
C3—C2—H2	120.1	C15—C14—H14	120.4
C1—C2—H2	120.1	C13—C14—H14	120.4
C2—C3—C4	119.4 (3)	C14—C15—C10	121.4 (3)
C2—C3—H3	120.3	C14—C15—H15	119.3
C4—C3—H3	120.3	C10—C15—H15	119.3
C9—C4—C5	118.2 (3)	C12—C16—H16A	109.5
C9—C4—C3	117.0 (3)	C12—C16—H16B	109.5
C5—C4—C3	124.8 (3)	H16A—C16—H16B	109.5
C6—C5—C4	120.3 (3)	C12—C16—H16C	109.5
C6—C5—H5	119.8	H16A—C16—H16C	109.5
C4—C5—H5	119.8	H16B—C16—H16C	109.5
C5—C6—C7	121.6 (3)	Cu1—Cl1—Cu1^i^	96.97 (3)
C5—C6—H6	119.2	O1—Cu1—N2	90.72 (10)
C7—C6—H6	119.2	O1—Cu1—N1	173.25 (9)
C8—C7—C6	119.7 (3)	N2—Cu1—N1	82.56 (10)
C8—C7—H7	120.2	O1—Cu1—Cl1	92.28 (6)
C6—C7—H7	120.2	N2—Cu1—Cl1	161.09 (9)
C7—C8—C9	119.9 (3)	N1—Cu1—Cl1	94.27 (7)
C7—C8—N2	126.4 (3)	O1—Cu1—Cl1^ii^	95.81 (7)
C9—C8—N2	113.8 (3)	N2—Cu1—Cl1^ii^	101.29 (8)
N1—C9—C8	117.1 (3)	N1—Cu1—Cl1^ii^	85.00 (8)
N1—C9—C4	122.6 (3)	Cl1—Cu1—Cl1^ii^	96.97 (3)
C8—C9—C4	120.3 (3)	C1—N1—C9	118.0 (3)
N3—C10—C15	113.6 (3)	C1—N1—Cu1	129.8 (2)
N3—C10—C11	127.0 (3)	C9—N1—Cu1	112.1 (2)
C15—C10—C11	119.4 (3)	N3—N2—C8	114.6 (2)
O1—C11—C12	118.9 (3)	N3—N2—Cu1	131.0 (2)
O1—C11—C10	122.9 (3)	C8—N2—Cu1	114.40 (19)
C12—C11—C10	118.2 (3)	N2—N3—C10	120.5 (3)
C13—C12—C11	119.1 (3)	C11—O1—Cu1	127.15 (19)
C13—C12—C16	121.0 (3)	C13—O2—H2A	107 (3)
C11—C12—C16	119.8 (3)	H3W—O3—H4W	114 (5)

Symmetry codes: (i) *x*−1, *y*, *z*; (ii) *x*+1, *y*, *z*.

### Hydrogen-bond geometry (Å, º)

**Table:**

*D*—H···*A*	*D*—H	H···*A*	*D*···*A*	*D*—H···*A*
C14—H14···O3	0.95	2.65	3.318 (4)	128
O2—H2*A*···O3	0.82 (4)	1.87 (4)	2.686 (4)	172 (4)
O3—H3*W*···Cl1^iii^	0.70 (4)	2.45 (4)	3.104 (3)	157 (5)
O3—H4*W*···O1^iv^	0.89 (6)	2.20 (6)	2.911 (4)	136 (4)

Symmetry codes: (iii) *x*+1/2, −*y*+3/2, *z*−1/2; (iv) *x*−1/2, −*y*+3/2, *z*−1/2.
